# Wound Healing Potential of Herbal Hydrogel Formulations of *Cedrus brevifolia* Extracts in Mice

**DOI:** 10.3390/gels10110750

**Published:** 2024-11-19

**Authors:** Georgia Lyggitsou, Christina Barda, Maria Anagnostou, Andreas Douros, Dimitra Statha, Christina Karampasi, Anastasia Ioanna Papantonaki, Ioannis Svoliantopoulos, Ioannis Sfiniadakis, Andreas Vitsos, Helen Skaltsa, Michail Ch. Rallis

**Affiliations:** 1 Section of Pharmaceutical Technology, Department of Pharmacy, National and Kapodistrian University of Athens, Panepistimiopolis Zografou, 15784 Athens, Greece; 2 Section of Pharmacognosy and Chemistry of Natural Products, Department of Pharmacy, National and Kapodistrian University of Athens, Panepistimiopolis Zografou, 15771 Athens, Greece; 3 Pathologoanatomic Laboratory, Athens Naval Hospital, 11521 Athens, Greece

**Keywords:** *Cedrus brevifolia*, plant extract, hydrogel, wound healing, in vivo, SKH-hr2, FT-IR, NMR

## Abstract

Wound healing stands as a paramount therapeutic pursuit, imposing significant challenges on healthcare, particularly for vulnerable populations. *Cedrus brevifolia*, a species endemic to Cyprus, thrives in the Tripylos region, commonly known as Cedar Valley, within the Paphos forest. Despite its endemism, this species exhibits negligible genetic divergence from its Mediterranean related species. This study aims to investigate the potential of *C. brevifolia* resin and bark extracts in promoting wound healing in a mouse model. Previous in vitro investigations have elucidated the antioxidant and anti-inflammatory potential of extracts and isolates derived from the title plant, warranting further exploration in an in vivo setting. This experimental design employed 40 male SKH-hr2 black and brown mice aged 2–4 months. Wounds measuring 1 cm^2^ were meticulously induced in the anesthetized mice and the potential healing effect of the herbal hydrogel formulations was evaluated. The healing potential of the *C. brevifolia* extracts was rigorously assessed through the daily application of gel formulations containing resin concentrations of 5% and 10% *w*/*w*, alongside sapwood and heartwood extracts at concentrations of 0.5% and 1% *w*/*w*. The evaluation of the treatments encompassed a multifaceted approach, incorporating clinical observations, skin biophysical parameter assessments utilizing an Antera 3D camera, and FT-IR spectroscopy, in addition to histopathological examination. The chemical compositions were also investigated through NMR and bio-guided isolation. The most prominent herbal hydrogel preparation proved to be the 10% resin, followed by the sapwood at 1%. The chemical analysis unveiled abietic acid, manool, and lariciresinol derivatives that potentially contributed to the observed results. Bridging the gap between in vitro observations and in vivo outcomes attempts to shed light on the potential therapeutic benefits of *C. brevifolia* hydrogels in wound care.

## 1. Introduction

Wound healing remains a critical therapeutic challenge in modern healthcare systems worldwide, particularly affecting vulnerable populations with chronic wounds, various comorbidities, and the elderly [1,2,3,4]. Non-healing wounds can lead to severe complications, including increased morbidity and, in extreme cases, mortality [2,3,4]. Chronic wounds, notably, can cause significant health issues and a diminished quality of life. These persistent and complex wounds often resist conventional treatments, underscoring the urgent need for innovative and effective wound healing therapies [4,5,6,7,8,9].

The wound healing process involves a complex, multi-step biological event, comprising haemostasis, inflammation, proliferation, and remodeling [1,2,3,6]. Initially, haemostasis rapidly stops bleeding through blood clot formation, which is followed by the inflammatory phase, where immune responses remove pathogens and debris, setting the stage for healing [3,4]. Subsequently, during the proliferation phase, new tissue formation occurs, including the production of granulation tissue and re-epithelialization [2,3,4]. Finally, the remodeling phase reorganizes and strengthens the newly formed tissue, restoring the skin’s integrity and functionality [3,4]. This intricate sequence is crucial for effective wound repair, but it can be disrupted by various factors, leading to delayed or impaired healing [2,3,4,5].

Having confirmed their effectiveness, hydrogels are among the most widely used formulations for wound treatment. One of their key features is that they contain over 90% water, which helps create a moist environment around the wound which is a crucial factor for promoting healing. This is due to its structure consisting of three-dimensional hydrophilic polymer networks that can absorb and maintain a significant amount of water. This capability not only retains moisture but also ensures that the hydrogels are biocompatible, meaning they are safe and well-tolerated by the body. This results in a soft, elastic, and porous structure that aligns well with biological tissues, making them suitable for use in wound care. They significantly accelerate two critical processes in wound repair, granulation, which involves the formation of new tissue, and epithelialization, the process of skin regrowth [9,10].

In parallel, the potential of plant-derived extracts as alternative therapeutic agents for wound healing has gained considerable attention [1,5,6,7,8]. Plants have been traditionally used for their medicinal properties, and recent scientific advancements have begun to elucidate the mechanisms behind their efficacy [1,2,3,6,8]. Among plant-derived candidates, the *Cedrus* genus stands out for its potential in this domain among others. Historically utilized in traditional medicine since ancient times for its antiseptic and healing properties, it continues to be important in modern therapy, particularly for wound care and inflammation management [7,11,12,13]. *Cedrus brevifolia* (Hook.f.) Elwes & A. Henry, commonly known as Cyprus Cedar, is endemic to the Tripylos region of Cyprus, thriving in Cedar Valley within the Paphos forest, and exhibits minimal genetic divergence from its Mediterranean counterparts. Previous in vitro studies have demonstrated the antioxidant and anti-inflammatory properties of extracts and isolated compounds from *C. brevifolia*, suggesting potential benefits for wound healing [11,13,14].

The main hypothesis of this study is that by combining the advantageous characteristics of gels with the pharmacological potential of plant extracts the wound healing outcomes will be significantly enhanced. This combination is emerging as a promising formula due to its ability to provide a controlled release of active compounds, enhance hydration, and create a protective barrier over wounds [4,5,9,10].

To address this, the present study aims to evaluate the wound healing efficacy of *C. brevifolia* resin and bark extracts in a mice model, representing a crucial step in bridging the gap between in vitro efficacy and in vivo application, potentially advancing Cedrus spp. as an alternative natural therapy for wound care. Through rigorous experimental design and evaluation, this study elucidates the potential of *C. brevifolia* extracts formulated into hydrogels for enhancing wound healing, thereby contributing to the broader field of wound management and therapy.

## 2. Results and Discussion

### 2.1. Phytochemical Analysis

Previous chemical investigations of the plant materials under study, as detailed by Douros et al. [12,14], provided an extensive characterization of their phytochemical profile with its representative compound groups being flavonoids, lignans, tannins, and terpenes, among others. In the current study, freshly prepared extracts and resin powder were formulated into gels for the in vivo evaluation of their therapeutic potential in mice. The chemical fingerprinting of the obtained extracts were initially traced dawn by ^1^H NMR (Figure 1) and 2D NMR experiments (see Figures S1–S13).

The ^1^H NMR spectra revealed related chemical profiles between the sapwood and heartwood H_2_O and EtOH extracts in terms of compound accumulation. The peaks observed in the region of 8.50–5.50 ppm indicate variations in the phenolic content between the two plant parts [15], with notable differences in the specific compound groups. The heartwood contained a higher concentration of abietane-type diterpenoids, tannins, and phenolic compounds [11].

The most prominent plant material, resin, displayed a unique NMR profile, predominantly featuring aromatic signals, attributable to lignans, with characteristic peaks appearing between *δ*_H_ 7.5 and 5.5, indicative of lariciresinol derivatives. In the broader aromatic region, signals attributed to phenolic compounds such as caffeic acid derivatives are present. Additionally, manool derivatives (manool and 3-hydroxy-manool) and abietic acid derivatives (including dehydroabietic acid) appear in the region of *δ*_H_ 6.00–1.00.

In order to verify the above observations, fractionations of the *C. brevifolia* wood and resin extracts was carried out. From the dichloromethane extract of the bark, one phenolic acid was isolated and identified, namely (*E*)-ferulic acid (**1**), which was the main secondary metabolite of the extract. Moreover, compound 1 was also isolated from the methanol extract of the bark, alongside syringic acid (**2**), quercetin (**3**), eriodictyol (**4**), and cilicione-b (**5**). From the cyclohexane extract of the resin, three diterpenes were isolated and identified, dehydroabietic acid (**6**), manool (**7**), and 3β-hydroxy-manool (**8**). In addition, the ethanol extract of the resin yielded two lignans, (+) lariciresinol (**9**) and (+) 9-acetyl lariciresinol (**10**); one phenolic compound, (*E*)-caffeic acid (**11**); and, while from the ethanol: H_2_O (1:1) of the resin, (+) lariciresinol (**9**) was again isolated.

To provide a more comprehensive understanding, additional wound healing properties of the *C. brevifolia* isolates were considered. According to previous studies, abietic acid derivatives exhibit anti-inflammatory and wound healing effects both in vitro and in vivo [16]. Lignans are known for their anti-inflammatory and antimicrobial properties while (+) lariciresinol is known for its antioxidant potential [17] and has been correlated to positive influences on scar tissue based on its observed effects on human skin fibroblasts [18]. Meanwhile, caffeic acid derivatives are well known for their anti-inflammatory and wound healing effects [19]. In contrast, there is a lack of information regarding the wound healing potential of manool derivatives, underscoring the need for further studies to explore their therapeutic contribution.

The potential of such isolates in wound treatment has been highlighted in numerous studies, particularly those showcasing their antioxidant, anti-inflammatory, and antimicrobial effects. Compounds such as terpenes and polyphenols are noted for their ability to interact at different stages of the wound healing process [20].

### 2.2. Formulation Development and Evaluation

The hydrogel formulations in this study were carefully designed based on previous research and the availability of raw materials. Carbopol 940, a well-established gelling agent commonly used in topical pharmaceutical and cosmetic applications, was selected as the primary medium. Its ability to produce clear, stable hydrogels with a high viscosity at low concentrations makes it an ideal carrier for the active extracts of *C. brevifolia*. In this study, Carbopol 940 played a critical role in ensuring the uniform dispersion of the resin, sapwood, and heartwood extracts, maintaining their bioactivity throughout the experimental period [21,22]. Additionally, its use facilitated the controlled release of the extract ingredients, enhancing their availability and efficacy at the wound site. Propylene glycol was also employed in the formulation to dissolve the materials effectively. However, despite its advantages, it is important to acknowledge that Carbopol 940 can, in some cases, cause skin irritation in sensitive individuals. This issue is an important consideration for long-term clinical applications, and further studies should aim to evaluate the dermal safety profile of this formulation in human subjects. The extracts of *C. brevifolia*, including resin, sapwood, and heartwood, were incorporated into the hydrogels based on their well-documented anti-inflammatory, antioxidant, and wound-healing properties [5,10,11,13]. The resin was included at concentrations of 5% and 10%. The sapwood and heartwood extracts were added at 0.5% and 1%, respectively. The doses for these formulations were selected based on prior experience and the availability of raw materials, with careful consideration given to ensuring that the concentrations were sufficient to provide therapeutic efficacy without inducing toxicity.

### 2.3. Experimental Evaluation

**Figure 2:**
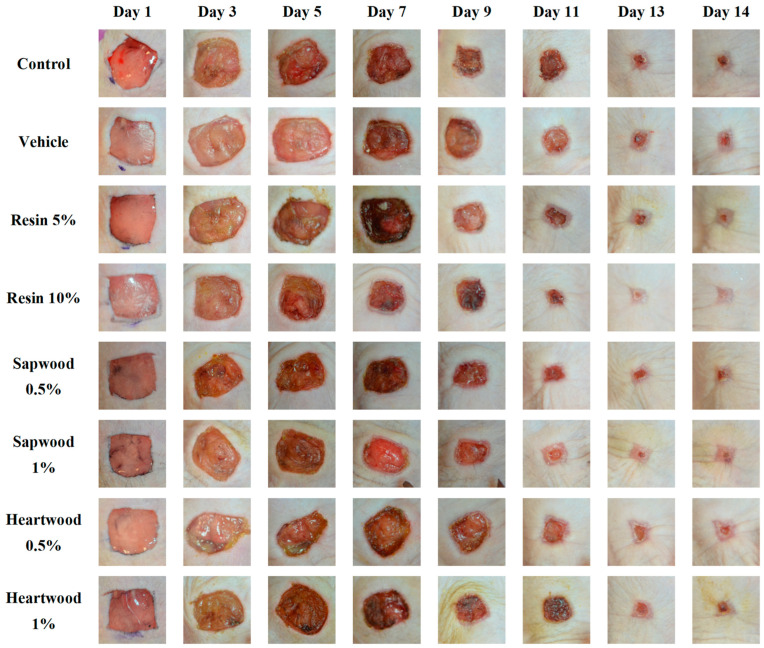
Photodocumentation throughout the experiment (days 1, 3, 5, 7, 9, 11, 13, and 14) displays the assessment of wound healing across eight groups: the control, the vehicle, and the groups that received a gel containing resin or *C. brevifolia* extracts (resin 5% and 10%, sapwood and heartwood extracts 0.5% and 1%, respectively).

**Figure 3:**
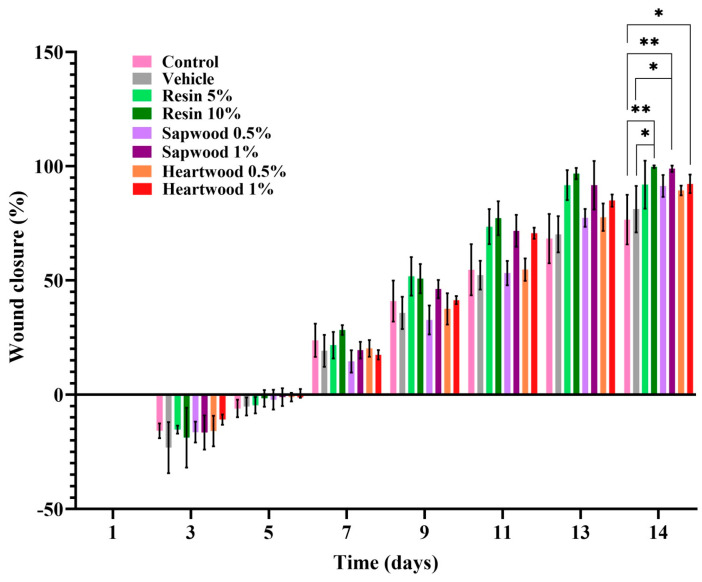
Wound healing closure (%) in relation to time. Statistical significance: control vs. resin 10% and sapwood extract 1% (*p* < 0.01; **), heartwood extract 1% (*p* < 0.05), and vehicle vs. resin 10% and sapwood extract 1% (*p* < 0.05; *).

Images depicting the wound healing process of SKH-hr2 hairless mouse skin were taken at multiple time points over a 14-day period (Figure 2). The results demonstrated that the groups treated with gels containing 10% resin and 1% sapwood extract achieved mean wound closures of 99.76% and 98.87%, respectively, significantly outperforming the vehicle (81.18%, *p* < 0.05) and the control (76.59%, *p* < 0.01) groups (Figure 3). The gel with 1% heartwood extract exhibited significant wound healing, achieving a closure of 92.25%, indicating a superior healing efficacy compared to the control group (*p* < 0.05) (Figure 3).

The negative values observed in Figure 2 during the first few days reflect the natural progression of the wound healing process. Initially, skin injuries often deepen and expand in surface area, a phenomenon that can persist for up to 5–6 days post-injury. This initial worsening phase is common and precedes the subsequent healing process [23].

In contrast, the untreated group and the group treated with a gel containing only excipients (control and vehicle groups) displayed incomplete healing in most cases. These findings for the control group align with similar studies [24,25], where wound closure was not fully achieved until the 16th day, suggesting a delayed healing process in the absence of treatment.

The outcomes indicate that the gel formulations, particularly those containing resin and sapwood extracts, significantly enhanced the wound healing compared to the control and vehicle treatments.

### 2.4. Histopathological Evaluation

**Figure 4:**
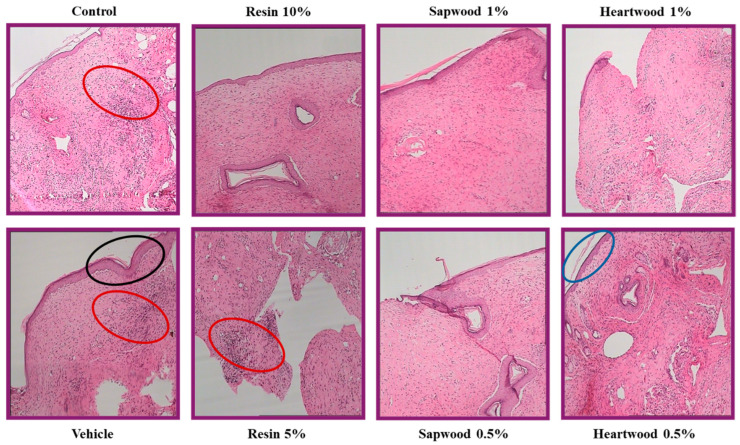
Skin sections at the end of the experimental procedure, stained with hematoxylin-eosin (100×). Representative annotation: the red ellipse depicts intense inflammatory elements; the back ellipse depicts parakeratosis; and the blue ellipse depicts epidermal hyperplasia.

A histopathological analysis of the representative skin biopsies revealed distinct differences in the tissue responses among the various treatment groups. The scoring of the histopathological features, such as inflammation, hyperkeratosis, and epidermal hyperplasia, provided a detailed understanding of the tissue healing and response mechanisms. For the control group (Score: 11), the biopsies exhibited intense inflammatory infiltration characterized by the presence of lymphocytes, plasma cells, and polymorphonuclear leukocytes. Mild epidermal hyperplasia was also observed (Figure 4, Table 1). Regarding the vehicle group (Score: 11), the vehicle-treated group showed marked hyperkeratosis accompanied by dense inflammatory infiltration (Figure 4). For the resin 10% (Score: 1), the treatment with 10% resin resulted in minimal inflammatory elements, with no significant hyperkeratosis in the overlying epidermis (Figure 4), while the resin 5% group (Score: 10) was associated with intense inflammatory elements throughout the entire dermal thickness (Figure 4), giving additional evidence regarding the therapeutic dose range. The sapwood extract groups, 0.5% and 1% (Score: 10 and 9, respectively), both demonstrated milder inflammatory infiltration in the healing area, with occasional clusters of a small number of inflammatory cells. The 1% sapwood treatment showed a slightly better healing profile with less inflammation compared to the 0.5% treatment (Figure 4). The heartwood extract groups, at concentrations of 0.5% and 1% (Scores: 9 and 8, respectively), exhibited inflammatory elements throughout the full thickness of the dermis. However, in contrast to the sapwood extract and 5% resin groups, no evidence of parakeratosis was detected.

These findings illustrate that the resin and sapwood extracts, particularly at higher concentrations, contributed to varying degrees of inflammation and tissue response. The resin 10% group showed the best healing outcomes with minimal inflammation, whereas the control and vehicle groups displayed significant inflammatory responses and epidermal changes. The sapwood treatments provided moderate healing with a relatively lower inflammatory profile, indicating a dose-dependent effect on skin recovery and inflammation.

The histological assessments underscore the potential efficacy of these plant extracts in modulating inflammation and promoting skin healing, which could be valuable for developing therapeutic applications.

### 2.5. Evaluation of Skin Parameters: Transepidermal Water Loss, Hydration, and Hemoglobin Measurements

**Figure 5:**
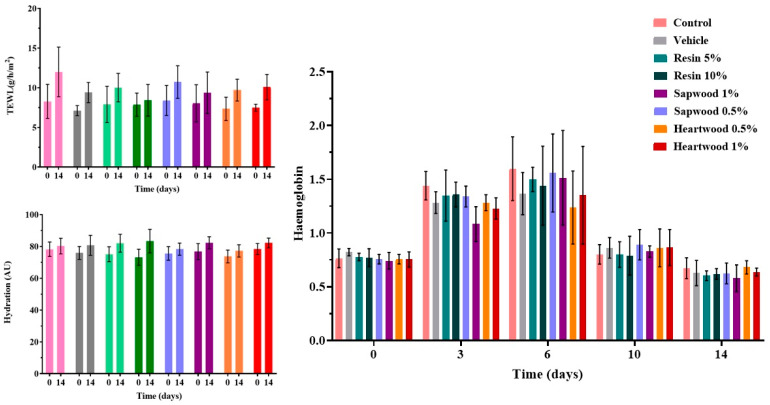
TEWL, hydration, and hemoglobin measurements, taken before the wound infliction and on the last day of the experiment.

Regarding transepidermal water loss (TEWL) (Figure 5), the graph illustrates an increase in the transepidermal water loss on the final day of the experiment across all the groups. This increase is expected and is in accordance with previous studies [24,25]. The wound healing process often compromises the skin barrier, leading to a scar with enhanced passive water loss through the epidermis [26]. The rejuvenation of the skin barrier appears to be more pronounced in the group treated with the 10% resin gel, as evidenced by the lack of statistically significant differences in the TEWL between day 1 and day 14, indicating prompt recovery. In contrast, the control, the vehicle, and the sapwood 0.5% and heartwood 1% groups exhibited substantial increases in the TEWL values, reflecting an affected skin barrier (with statistically significant differences *p* < 0.05).

The hydration levels on days 1 and 14 showed a slight significant increase in the cases of the resin 5%, sapwood 0.5 and 1%, and heartwood 1%, potentially due to the use of the dressing gels applied on the wounds. Similar results were obtained previously by Vitsos et al. [25].

The hemoglobin levels increased during the initial days of healing across all the groups (Figure 5). This is in accordance with the experimental observation of enhanced inflammation during the first week after the wound induction. The increase in the hemoglobin levels during the initial days of healing across all the groups (Figure 5) aligns with the experimental observations in the mice which indicate heightened inflammation in the first week following the wound induction. This inflammatory response is a normal part of the wound healing process, wherein increased blood flow occurs to initiate tissue repair. The elevated hemoglobin levels reflect the body’s effort to deliver more oxygen and nutrients to the healing tissue, supporting the inflammatory phase of wound healing. The significant decrease in the hemoglobin levels observed on day 10 in all the cases aligns with the clinical observations of reduced inflammation. This reduction is indicative of the transition from the inflammatory phase to the proliferative phase of wound healing [27].

### 2.6. ATR- FT-IR Spectroscopic Analysis

**Figure 6:**
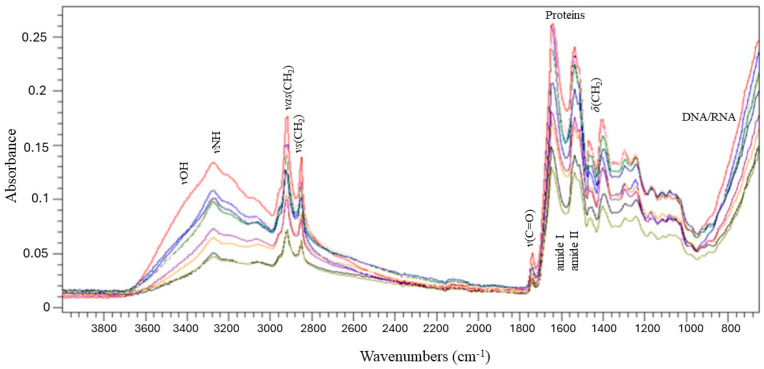
Represents a comparison of normalized and averaged FT-IR spectra on day 14 post-lesion for the healthy skin (blue), control group (untreated mice; red), vehicle control group (mice treated only with excipients; navy blue), 5% *w*/*w* (black) and 10% *w*/*w* (pink) resin gels, 0.5% *w*/*w* (purple) and 1% *w*/*w* (olive) sapwood extract gels, and 0.5% *w*/*w* (green) and 1% *w*/*w* (orange) heartwood extract gels. Changes in the band frequency, intensity, and shape were observed across the spectral regions 3700–3000 cm^−1^, 3000–2850 cm^−1^, and 1800–800 cm^−1^.

Using a portable ATR-FT-IR spectrometer, spectra were recorded from the mice skin to gain insights into the scar and wound skin quality. This approach allowed us to capture the real-time, non-invasive molecular fingerprints of the skin. Specific absorbance bands associated with lipids, proteins, carbonyls, and water were identified, providing a thorough profile of the scar and wound skin quality in relation to normal skin. Figure 6 presents a comparison of normalized and averaged FT-IR spectra on day 14 post-lesion for the healthy skin, wounded skin without treatment (control; in red), and wounded skin treated either with the vehicle ingredients or the gel formulations of the *C. brevifolia* extracts. Changes in the band frequency, intensity, and shape were observed across the spectral regions 3700–3000 cm^−1^, 3000–2850 cm^−1^, and 1800–800 cm^−1^, especially in the untreated group (red) and the healthy skin (blue).

Changes in the band frequency, intensity, and shape were observed in the spectral regions 3700–3000 cm^−1^, 3000–2850 cm^−1^, and 1800–800 cm^−1^. Lipid-related peaks (2850 cm^−1^, 2920 cm^−1^) and the 1737 cm^−1^ band indicated higher absorbance and oxidation in the untreated group than in the healthy group (and treated skin). Amide I (ca1650 cm^−1^) and Amide II (ca1550 cm^−1^) bands showed protein structure changes, with collagen peaks (1235 cm^−1^, 1330 cm^−1^) also being more intense in the untreated group vs. the healthy skin. The protein changes indicate that the skin had not yet returned to its normal state and had remained in the natural remodeling phase. Lastly, the 1140–1000 cm^−1^ region revealed nucleic acid and carbohydrate changes, indicating differences in the metabolic activity [28,29,30].

This ATR-FT-IR spectroscopy analysis was used to track spectral changes during skin wound healing in mice, providing key data in a growing field. It was characterized by the distinct profile of the control group (the untreated mice vs. the other treatment groups). The recent literature highlights ATR-FT-IR’s increasing role in real-time wound analysis with functional groups and band shifts indicating variations in skin composition and metabolism. Differences in band intensity and position from 4000 to 800 cm^−1^ suggest distinct biochemical processes. Nevertheless, further research is needed to clarify these spectral changes, refine analysis methods, and integrate complementary validation techniques. It is worth noting that the use of ATR-FTIR spectroscopy is a novel practice that enables the capture of spectral changes in mice skin, providing additional data for understanding skin progress during wound healing. These findings lay the groundwork for real-time ATR-FTIR applications in research, potentially enhancing diagnostics and treatment monitoring, especially in pre-clinical studies.

## 3. Conclusions

This study presents a comprehensive analysis through clinical, histopathological, biophysical, and FT-IR evaluations, highlighting the wound healing efficacy of hydrogels formulated with *C. brevifolia* resin material and bark extract. Our results reinforce the transition from in vitro findings to in vivo outcomes, confirming the potential of these formulations in practical wound care. Specifically, the resin-containing hydrogels, particularly at a 10% concentration, followed closely by the sapwood extract, demonstrated significant therapeutic potential. These findings suggest that further studies should investigate the scalability and clinical applicability of such formulations in human models, given their superior performance in enhancing wound closure and tissue regeneration. The bioactive compounds identified, including abietic acid, lariciresinol derivatives, and manool, with known anti-inflammatory and wound healing properties, likely contribute to the observed effects. However, the precise mechanisms of action and the individual contributions of each compound warrant further exploration. In particular, future research should consider alternative formulations, such as isolated compounds or synergistic blends, to optimize therapeutic outcomes and validate the mechanisms driving wound healing efficacy.

## 4. Materials and Methods

### 4.1. Reagents, Raw Materials, and Instrumentation

Ethanol 99% (Merck, Darmstadt, Germany), propylene glycol (Cellco Chemicals SA; Mandra, Greece), Carbopol 940 (Cellco Chemicals SA; Mandra, Grecce), triethanolamine (Merck; Darmstadt, Germany), ddH_2_O, ketamine 100 mg/Kg (Narketan 10, 100 mg/mL Vetoquinol SA, Lure–Cedex, France), xylazine 7 mg/Kg (Xylapan 20 mg/mL, Libourne, France), Fixomull Stretch Self Adhesive Gauze (BSN Medical, Hamburg, Germany), Medicomp sterile gauze (Hartmann, Heidenheim, Germany), and paracetamol (Vianex, Athens, Greece) were used.

^1^H, ^13^C, and 2D-NMR spectra were recorded on a Bruker DRX 400 (Bruker BioSpin GmbH, Silberstetten, Germany) and spectrometers at 295 K. Chemical shifts were reported in ppm (δ) using the residual solvent signal (δH 3.31 in ^1^H and δC 49.0 in ^13^C, CD_3_OD) as a reference. Vacuum liquid chromatography (VLC) was performed on silica gel (Merck: 43–63 m) (Merck KGaA, Darmstadt, Germany), and column chromatography (CC) was performed on silica gel 60H (SDS: 40–63 m) and a Sephadex LH 20 (Pharmacia, Uppsala, Sweden). Gradient elution with the solvent’s mixtures was used as an indicator in each case. Thin-layer chromatography plates were pre-coated with silica gel 60 (Merck, Art. 5721). Fractions monitoring to follow the separation was performed by thin-layer chromatography (TLC) on silica gel 60 F254 (Merck, Art. 5554; Darmstadt, Germany) and cellulose (Merck Art. 5552; Darmstadt, Germany). Compounds were detected using UV absorbance (254 and 365 nm). Vanillin/sulphuric acid reagent (vanillin 5% in H_2_SO_4_/MeOH 1:1) and β-aminoethylester of diphenyl boric acid 1% in MeOH were used for detection in the TLC. Analytical solvents were obtained from Panreac Quimica SA (Barcelone, Spain, Italy), while deuterated solvents were purchased from Merck, KGaA (Darmstadt, Germany).

#### 4.1.1. Plant Material

*C. brevifolia* wood cut down from Cedar valley near Paphos (Cyprus) in July 2017 and the resin were provided by the Department of Forests, Ministry of Agriculture, Natural Resources and Environment, Nicosia, Cyprus. The plant material was authenticated by Mr. Konstantinos Nikolaou.

#### 4.1.2. Plant Extract Preparation for the In Vivo Experiment

The sapwood of the *C. brevifolia* (7.65 g) was extracted with ethanol (EtOH) and water (H_2_O), successively (DER: 5:1), three times (for 24 h each time) at room temperature. The obtained extracts were concentrated to dryness and yielding residues of 0.0464 g and 0.0156 g. The heartwood (7.97 g) was similarly extracted and afforded residues of 0.5103 g and 0.0910 g, respectively.

Following the extractions, the ethanol and water extracts for each plant material were combined based on their ^1^H-NMR fingerprints. This analysis ensured that the extracts were optimally combined to maximize the presence of the desired compounds. This process resulted in two hydroalcoholic extracts with final amounts of 0.0620 g and 0.6013 g, respectively.

#### 4.1.3. Isolation and Structure Elucidation

The *C. brevifolia* wood (sapwood and heartwood) was extracted with dichloromethane and methanol, successively. A total of 1.0 g of the dichloromethane extract was fractionated using silica gel column chromatography with cyclohexane:EtOAc mixtures of increasing polarities (100:0–0:100, 75 fractions). The fractions eluted with cyclohexane:EtOAc 93:7 and 90:10 were identified as (*E*)-ferulic acid (1, 147.7 mg). The methanol extract of the wood (5.13 g) was subjected to vacuum column chromatography (VLC) (6.0 × 5.5 cm) and the eluted mixtures of CyHex:EtOAc:MeOH (100:0:0–0:100:100; 10 fractions of 500 mL for each one). The fraction eluted with CyHex:EtOAc 50:50 was further subject to CC over Sephadex LH −20 (25.0 × 1.5 cm) with MeOH 100% and yielded syringic acid (2, 1.2 mg), quercetin (3, 4.3 mg), eriodictyol (4, 3.3 mg), and cilicione-b (5, 9.0 mg). The fraction eluted with CyHex:EtOAc 90:10 afforded (*E*)-ferulic acid (1, 17.9 mg). The resin was extracted with cyclohexane, EtOH, and EtOH: H_2_O 1:1, successively. Cyclohexane extract (0.43 g) was subjected to CC over silica gel (8 × 1.5 cm) with cyclohexane:EtOAc of increasing polarities (100:0–30:70, 40 fractions). The fractions eluted with cyclohexane:EtOAc 95:5, 90:10, 60:40 yielded dehydroabietic acid (6, 1.2 mg), manool (7, 186.9 mg), and 3β-hydroxy-manool (8, 1.8 mg), respectively. The ethanol extract (7.2 g) was subjected to vacuum column chromatography (VLC) (9.0 × 5.5 cm) and eluted mixtures of CyHex:EtOAc (100:0–0:100; 10 fractions of 500 mL for each one). The fractions eluted with CyHex:EtOAc 70:30, 60:40, and 50:50 afforded (+) lariciresinol (9, 2.7 mg), (+) 9-acetyl lariciresinol (10, 110.7 mg), and (*E*)-caffeic acid (11, 7.4 mg), respectively. Finally, the EtOH: H_2_O 1:1 extract (0.37 g), similarly fractionated, yielded (+) lariciresinol (9, 27.9 mg) as the main compound.

#### 4.1.4. Preparation of the Gel Formulations

After pulverizing a portion of the resin into a fine powder using a mortar and pestle and conducting solubility tests of the resin powder in propylene glycol at various concentrations, 5% *w*/*w* and 10% *w*/*w* resin gels were formulated. These gels consisted of distilled water, as an aqueous carrier of the gel, Carbopol 940 2% *w*/*w*, as a gelling agent, propylene glycol 10% *w*/*w*, as a solvent of the resin and humectant agent, and triethanolamine, for pH regulation (6.5). Carbopol 940 is a harmless polyacrylic acid that binds polar compounds such as water and alcohols and improves the adhesion and spreadability of hydrogels, contributing to their good skin permeability [21,22].

Using the same excipients and proportions, gels incorporating the sapwood and heartwood extracts were obtained (Table 2). 

### 4.2. In Vivo Study Design and Animals

All the procedures were performed in accordance with the European Communities Council Directive 2010/63/EU of 22 September 2010, and the study protocols conformed to the ARRIVE guidelines [25]. A total of forty male SKH-hr2 hairless mice, aged 2–4 months, from the breeding stock of the Department of Pharmacy Small Animal Laboratory (EL 25 BIO-BR 06) were used in this study. The sample size was selected based on similar published pilot studies to ensure adequate statistical relevance [26]. The animal housing conditions were maintained at 23 ± 1 °C with a 25–55% relative humidity. The illumination cycle was controlled using yellow, fluorescent lights, providing a 12 h light/dark cycle. The mice had continuous access to a standard chow diet (Nuevo SA-Farma-Efyra Industrial and Commercial SA, Gerakas, Greece) and fresh water throughout the study.

Approval for the experimental procedures was granted by the National Peripheral Veterinary Authority (Protocol Number: 1612154/29.12.23) following an assessment by the Animal Protocols Evaluation Committee. Prior to the commencement of the experiment, a one-week acclimatization period was provided to all the animals.

The mice were categorized by age and then randomly allocated into eight groups of five animals each (n = 5) to ensure balanced age and weight distributions across all the groups. The experimental groups were defined as follows:

1. Control group (untreated mice).
2. Vehicle control group (mice treated with the gel vehicle containing only excipients, without any therapeutic agents).
3. Mice treated with 5% *w*/*w* resin gel.
4. Mice treated with 10% *w*/*w* resin gel.
5. Mice treated with 0.5% *w*/*w* sapwood extract gel.
6. Mice treated with 1% *w*/*w* sapwood extract gel.
7. Mice treated with 0.5% *w*/*w* heartwood extract gel.
8. Mice treated with 1% *w*/*w* heartwood extract gel.

### 4.3. Weight and Temperature Measurements

Animal welfare monitoring was performed by measuring the mice’s weight and temperature at both the start and conclusion of the experiment. The weight was measured using an electronic scale (KERN EHA 3000-0, KERN & SOHN GmbH, Balingen, Germany), while the temperature was assessed using a contactless infrared thermometer (NC150, Microlife, Windaus, Switzerland). To ensure accuracy, the temperature measurements were taken three times for each animal.

### 4.4. Wound Infliction and Maintenance 

On the first day of the study, all the mice were anesthetized using a combination of ketamine and xylazine, administered intraperitoneally at doses of 100 mg/kg for the ketamine (Narketan 10, 100 mg/mL, France) and 7 mg/kg for the xylazine (Xylapan 20 mg/mL, France). Following anesthesia, full-thickness wounds were created on the dorsal region using a 1 cm × 1 cm sterile surgical blade, and the wounds were standardized using sterile forceps to ensure uniformity across all the subjects.

Post-surgery, the mice were provided with water containing 1–2 mg/mL paracetamol for the first three days to manage the pain. Each wound was treated daily with the designated topical application according to the group assignment. For all the groups, the wounds were cleaned daily with sterile saline to remove debris and exudates, and any necrotic tissue was carefully debrided as necessary.

The dose of hydrogel was set at 5 mg/cm^2^ per application. This parameter was established to maintain a consistent dose throughout the experiment, ensuring a uniform exposure to the active ingredients regardless of changes in the wound size.

After each treatment, the wound was covered with a transparent film dressing followed by a layer of self-adhesive gauze (Fixomull Stretch, BSN Medical GmbH, Hambur, Germany) to protect the area. These dressings were changed every 24 h to maintain the wound hygiene and assess the healing progress.

The wound maintenance protocol ensured consistent treatment across all the groups and facilitated the accurate assessment of the healing properties of the formulations being tested.

### 4.5. Photodocumentation

The wounds from each group were photographed at time zero and every other day until the end of the experiment, using a Nikon D5100 digital camera (Nikon, Tokyo, Japan) equipped with an AF-S Micro Nikkor 60 mm f/2.8 G ED lens (Nikon, Tokyo, Japan). The camera was positioned at a fixed distance of 20 cm perpendicular to the subject. The photographs were digitized, and the wound area was measured at different time points during the healing process. The wound closure was determined by the following formula: Wound closure (%) = $\frac{A_{0}-A_{t}}{A_{0}}\times 100$, where A_0_ is the initial wound area and A_t_ is the wound area at different observation times.

Images were also captured using an Antera 3D camera (Miravex, Dublin, Ireland). The Antera 3D camera uses an optical method combined with a complex algorithm to capture images in three dimensions that allow the measurement of skin parameters with a high reliability. The software, version 2.11.5, was employed for the assessment of wound area (measured in mm^2^) and volume. 

### 4.6. Evaluation of Hydration

The hydration assessment focused on the stratum corneum, which is critical for maintaining the skin’s barrier function and appearance. Optimal water content in the stratum corneum ranges from 10% to 20% [31,32,33]. Skin is considered dry when the stratum corneum water content is below 10%, while levels above 16% can cause overhydration, disrupting the keratin layer’s compact structure [34].

The hydration levels were measured using the Corneometer^®^ CM 820 (Courage-Khazaka, Koln, Germany). This instrument quantifies hydration on a scale from 0 (no water content) to 120 (high water content), with the results expressed in “Corneometer units”. The Corneometer’s measurement is based on changes in capacitance due to skin surface hydration, affecting only the surface moisture with a minimal penetration depth. Each measurement is quick, lasting only one second, thereby minimizing any potential occlusion effects that could alter the results.

### 4.7. Evaluation of Transepidermal Water Loss (TEWL)

The assessment of the TEWL was performed using the Tewameter^®^ TM 240 (Courage-Khazaka, Cologne, Germany). This non-invasive instrument measures TEWL by employing an electrochemical detector system. It quantifies water loss indirectly through the use of two pairs of sensors that measure temperature and relative humidity within a hollow plastic cylinder, which is positioned vertically on the skin surface. These sensors are highly sensitive and allow for precise measurements. The data collected by the Tewameter^®^ are processed by the device’s microprocessor, which then expresses the results as the rate of water loss per unit of time and surface area, specifically in grams per hour per square meter (g/h/m^2^).

### 4.8. Collection of Skin Samples—Histopathological Evaluation

On the last day of the experiment (14th day), the animals were sacrificed by cervical dislocation. After that, biopsies of the skin area were obtained. The back skin wound area was removed, and tissue samples were fixed in 10% formalin and then embedded in paraffin-forming paraffin blocks, for histopathological evaluation. Sections were cut and stained with hematoxylin and eosin staining. The sections were examined under 100× magnification, and the extent of inflammation, edema, hyperkeratosis, wound thickness, ulceration, necrosis, and parakeratosis were blindly evaluated by a qualified anatomopathologist at the Pathology Laboratory of the Naval Hospital of Athens based on criteria outlined in Table 3.

### 4.9. Attenuated Total Reflection Fourier Transform Infrared (ATR-FT-IR) Spectroscopy

FT-IR spectra were recorded using a 4300 handheld FT-IR spectrometer (Agilent Technologies, Santa Clara, CA, USA) equipped with an attenuated total reflection (ATR) crystal. Data analysis was performed using OMNIC 7.1 software. The advantage of this non-destructive technique is that it enables researchers to record and analyze the skin surface in vivo, avoiding the use of biopsies and coloring, as in histopathology. The spectra were recorded on the 14th day post-wound both on the surface of the healthy skin, the formed scar, and on the surrounding irradiated skin.

### 4.10. Data Analysis

The data were examined to determine their distribution by using the Shapiro–Wilk test. The statistical significance between the groups was assessed using Student’s *t*-test and one-way analysis of variance (ANOVA), as well as their non-parametric equivalents, the Wilcoxon rank–sum test and the Kruskal–Wallis test, respectively. In all the cases, differences were deemed significant at *p* < 0.05. All analyses and graphical representations were performed utilizing GraphPad Prism 8.4.3 (GraphPad Software, Inc., San Diego, CA, USA).

### 4.11. Nuclear Magnetic Resonance (NMR) Spectroscopy

NMR spectroscopy was employed to characterize the chemical profiles of the extracts used in this study. NMR spectra were recorded using a Bruker DRX 400 (400 MHz for ^1^H-NMR, Bruker BioSpin, Rheinstetten, Germany) at a controlled temperature of 298 K. Chemical shifts were reported in parts per million (ppm, δ) relative to the solvent signals: 3.31 ppm for the ^1^H and 49.0 ppm for the ^13^C in methanol-d_4_ (CD_3_OD), and 7.24 ppm for the ^1^H and 77.0 ppm for the CDCl_3_.

To ensure a comprehensive chemical analysis, 1D and 2D NMR experiments, including correlation spectroscopy (COSY) and heteronuclear single-quantum correlation (HSQC,) were conducted using standard Bruker pulse programs. This approach facilitated the in-depth characterization of their chemical profiles and confirmed the presence of target compounds.

For additional validation, the 1D and 2D NMR spectra of the EtOH and H_2_O residues were also measured (see Supplementary Materials for details). The identification of metabolites was achieved by comparing the obtained chemical shifts and coupling constants with previously isolated compounds, as all the extracts analyzed in this study had been previously chemically characterized by Douros et al. [14]. The current NMR analysis verified the presence of the known compounds in each extract, ensuring the reliability and reproducibility of the chemical profiling data.

## Figures and Tables

**Figure 1:**
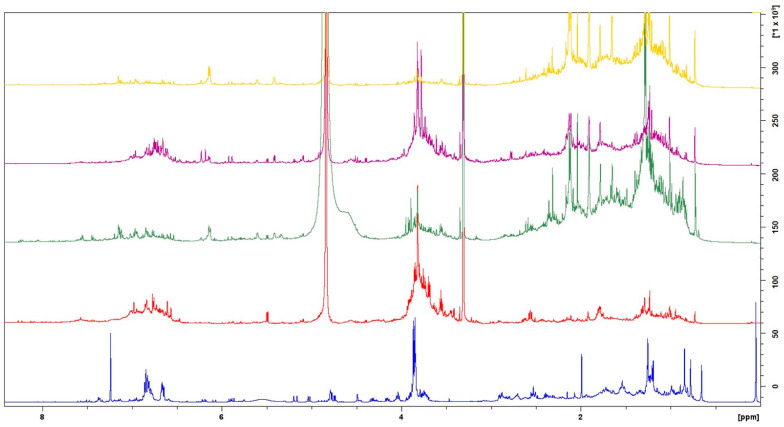
Overlaid ^1^H-NMR chemical fingerprints of *Cedrus brevifolia* resin (blue; in CDCl_3_), sapwood H_2_O (red; in MeOD), EtOH extracts (green; in MeOD), heartwood H_2_O (purple; in MeOD), and EtOH extracts (yellow; in MeOD).

**Table 1:** Histopathological assessment results.

Specimens’ 14th Day	Inflammation	Edema	Hyperkeratosis	Wound Depth	Ulceration	Necrosis	Parakeratosis	Score
Control	3	3	1	3	0	0	1	11
Vehicle	3	3	1	3	0	0	1	11
Resin 10%	0	0	1	0	0	0	0	1
Resin 5%	3	2	1	3	0	0	1	10
Sapwood extract 0.5%	3	2	1	3	0	0	1	10
Sapwood extract 1%	2	2	1	3	0	0	1	9
Heartwood extract 0.5%	2	2	1	3	0	0	1	9
Heartwood extract 1%	2	2	1	3	0	0	0	8

Scoring criteria for histopathological evaluation in Section 4.8.

**Table 2:** Composition of herbal gel formulation for wound healing activity.

	Quantity (% *w*/*w*)							
Groups	Control Group	Vehicle Group	Resin Gel 5%	Resin Gel 10%	Sapwood Extract Gel 0.5%	Sapwood Extract Gel 1%	Heartwood Extract Gel 0.5%	Heartwood Extract Gel 1%
Ingredients								
Plant part/extract	-	-	Resin 5%	Resin 10%	Sapwood extract 0.5%	Sapwood extract 1%	Heartwood extract 0.5%	Heartwood extract 1%
Carbopol 940	-	2%	2%	2%	2%	2%	2%	2%
Propylene glycol	-	10%	10%	10%	10%	10%	10%	10%
Triethanolamine	-	q.s.	q.s.	q.s.	q.s.	q.s.	q.s.	q.s.
Deionized water	-	q.s.	q.s.	q.s.	q.s.	q.s.	q.s.	q.s.

q.s.: quantum satis.

**Table 3:** Scoring criteria for histopathological evaluation.

Scoring Criteria for Histopathological Evaluation				
Inflammation	0 (absence)	1 (mild)	2 (moderate)	3 (heavy)
Edema	0 (absence)	1 (mild)	2 (moderate)	3 (heavy)
Hyperkeratosis	0 (absence)	1 (mild)	2 (moderate)	3 (heavy)
Wound thickness	0 (absence)	1 (superficial)	2 (moderate)	3 (total)
Ulceration	0 (absence)	1 (presence)		
Necrosis	0 (absence)	1 (presence)		
Parakeratosis	0 (absence)	1 (presence)		

## Data Availability

The data that support the findings of this study are available upon request.

## Supplementary Materials

The following supporting information can be downloaded at: https://www.mdpi.com/article/10.3390/gels10110750/s1, Figure S1: ^1^H-NMR (400 MHz, MeOD) spectrum of the ethanol extract of heartwood; Figure S2: ^1^H-^1^H COSY (MeOD) spectrum of the ethanol extract of heartwood; Figure S3: HSQC (MeOD) spectrum of spectrum of the ethanol extract of heartwood; Figure S4: ^1^H-NMR (400 MHz, MeOD) spectrum of the H_2_O extract of heartwood; Figure S5: ^1^H-^1^H COSY (MeOD) spectrum of the H_2_O extract of heartwood; Figure S6: HSQC (MeOD) spectrum of spectrum of the H_2_O extract of heartwood; Figure S7: ^1^H-NMR (400 MHz, MeOD) spectrum of the ethanol extract of sapwood; Figure S8: ^1^H-^1^H COSY (MeOD) spectrum of the ethanol extract of sapwood; Figure S9: HSQC (MeOD) spectrum of spectrum of the ethanol extract of sapwood; Figure S10: ^1^H-NMR (400 MHz, MeOD) spectrum of the H_2_O extract of sapwood; Figure S11: ^1^H-^1^H COSY (MeOD) spectrum of the H_2_O extract of sapwood; Figure S12: HSQC (MeOD) spectrum of spectrum of the H_2_O extract of sapwood; Figure S13: ^1^H-NMR (400 MHz, CDCl_3_) spectrum of the resin; Figure S14: A representative spectrum of normalized and averaged FT-IR spectra on day 14 post-lesion for healthy skin; Figure S15: A representative spectrum of normalized and averaged FT-IR spectra on day 14 post-lesion for the control group (untreated mice); Figure S16: A representative spectrum of normalized and averaged FT-IR spectra on day 14 post-lesion for the vehicle control group (mice treated only with excipients); Figure S17: A representative spectrum of normalized and averaged FT-IR spectra on day 14 post-lesion for the 5% *w*/*w* resin gel; Figure S18: A representative spectrum of normalized and averaged FT-IR spectra on day 14 post-lesion for the 10% *w*/*w* resin gel; Figure S19: A representative spectrum of normalized and averaged FT-IR spectra on day 14 post-lesion for the 1% *w*/*w* sapwood extract gel; Figure S20: A representative spectrum of normalized and averaged FT-IR spectra on day 14 post-lesion for the 1% *w*/*w* sapwood extract gel; Figure S21: A representative spectrum of normalized and averaged FT-IR spectra on day 14 post-lesion for the 0.5% *w*/*w* heartwood extract gel; Figure S22: A representative spectrum of normalized and averaged FT-IR spectra on day 14 post-lesion for the 1% *w*/*w* heartwood extract gel.
