# Supplementary material for: Wound Healing Potential of Herbal Hydrogel Formulations of Cedrus brevifolia Extracts in Mice
Source: Gels. 2024 Nov 19;10(11):750. doi: 10.3390/gels10110750 (PMC11593687; doi:10.3390/gels10110750)
Supplement: Supplementary file 1 [file gels-10-00750-s001.zip › gels-3121659-supplementary.pdf]

Supporting Information

Wound Healing Potential of Herbal Hydrogel Formulations of *Cedrus brevifolia* Extracts in Mice

Georgia Lyggitsou[1,2], Christina Barda[1,2], Maria Anagnostou[2], Andreas Douros[2], Dimitra Statha[1], Christina Karampasi[1], Anastasia – Ioanna Papantonaki[1], Ioannis Svoliantopoulos[1], Ioannis Sfiniadakis[3], Andreas Vitsos[1], Helen Skaltsa[2]*, Michail Ch. Rallis[1]*

[1]Section of Pharmaceutical Technology, Department of Pharmacy, National and Kapodistrian University of Athens, Panepistimiopolis Zografou, 15784 Athens, Greece

[2]Section of Pharmacognosy and Chemistry of Natural Products, Department of Pharmacy, National and Kapodistrian University of Athens, Panepistimiopolis Zografou, 15771 Athens, Greece

[3]Athens Naval Hospital, Pathologoanatomic Laboratory, 11521 Athens, Greece

*Correspondence: rallis@pharm.uoa.gr; skaltsa@pharm.uoa.gr

List of Contents

Figure S1. ^{1}H-NMR (400 MHz, MeOD) spectrum of the ethanol extract of heartwood 3
Figure S2. ^{1}H-^{1} H COSY (MeOD) spectrum of the ethanol extract of heartwood 3
Figure S3. HSQC (MeOD) spectrum of spectrum of the ethanol extract of heartwood 4
Figure S4. ^{1}H-NMR (400 MHz, MeOD) spectrum of the H_2O extract of heartwood 4
Figure S5. ^{1}H-^{1}H COSY (MeOD) spectrum of the H_2O extract of heartwood 5
Figure S6. HSQC (MeOD) spectrum of spectrum of the H_2O extract of heartwood 5
Figure S7. ^{1}H-NMR (400 MHz, MeOD) spectrum of the ethanol extract of sapwood 6
Figure S8. ^{1}H-^{1}H COSY spectrum of the ethanol extract of sapwood 6
Figure S9. HSQC (MeOD) spectrum of spectrum of the ethanol extract of sapwood 7
Figure S10. ^{1}H-NMR (400 MHz, MeOD) spectrum of the H_2O extract of sapwood 7
Figure S11. ^{1}H-^{1}H COSY (MeOD) spectrum of the H_2O extract of sapwood 8
Figure S12. HSQC (MeOD) spectrum of spectrum of the H_2O extract of sapwood 8
Figure S13. ^{1}H-NMR (400 MHz, $CDCl_3$) spectrum of the resin 9
Figure S14. A representative spectrum of normalized and averaged FT-IR spectra on day 14 post-lesion for healthy skin 9
Figure S15. A representative spectrum of normalized and averaged FT-IR spectra on day 14 post-lesion for the control group (untreated mice) 10
Figure S16. A representative spectrum of normalized and averaged FT-IR spectra on day 14 post-lesion for the vehicle control group (mice treated only with excipients 10
Figure S17. A representative spectrum of normalized and averaged FT-IR spectra on day 14 post-lesion for the 5% w/w resin gel 11
Figure S18. A representative spectrum of normalized and averaged FT-IR spectra on day 14 post-lesion for the 10% w/w resin gel 11
Figure S19. A representative spectrum of normalized and averaged FT-IR spectra on day 14 post-lesion for the 1% w/w sapwood extract gel 12
Figure S20. A representative spectrum of normalized and averaged FT-IR spectra on day 14 post-lesion for the 1% w/w sapwood extract gel 12
Figure S21. A representative spectrum of normalized and averaged FT-IR spectra on day 14 post-lesion for the 0.5% w/w heartwood extract gel 13
Figure S22. A representative spectrum of normalized and averaged FT-IR spectra on day 14 post-lesion for the 1% w/w heartwood extract gel 13

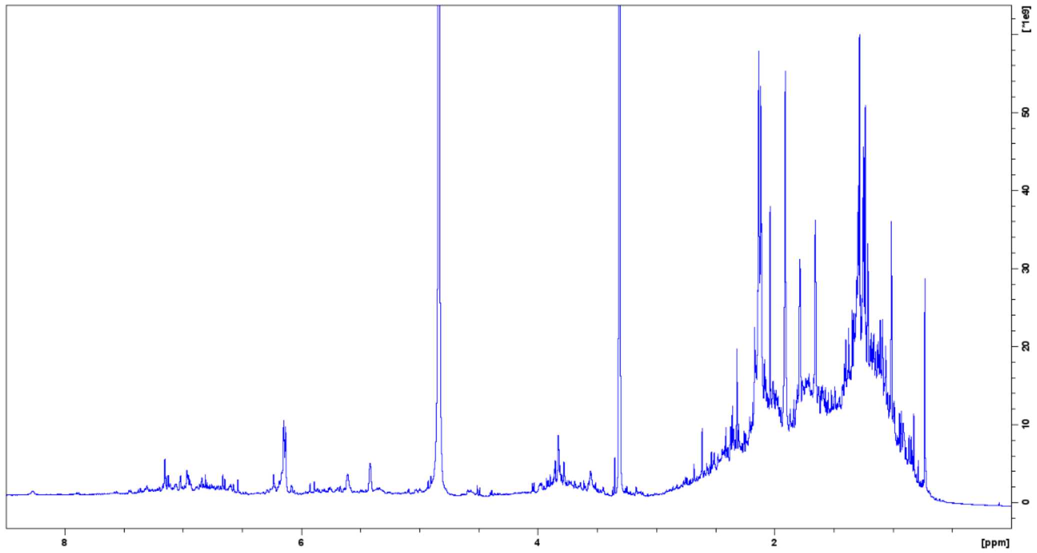

Figure S1. ^{1}H-NMR (400 MHz, MeOD) spectrum of the ethanol extract of heartwood

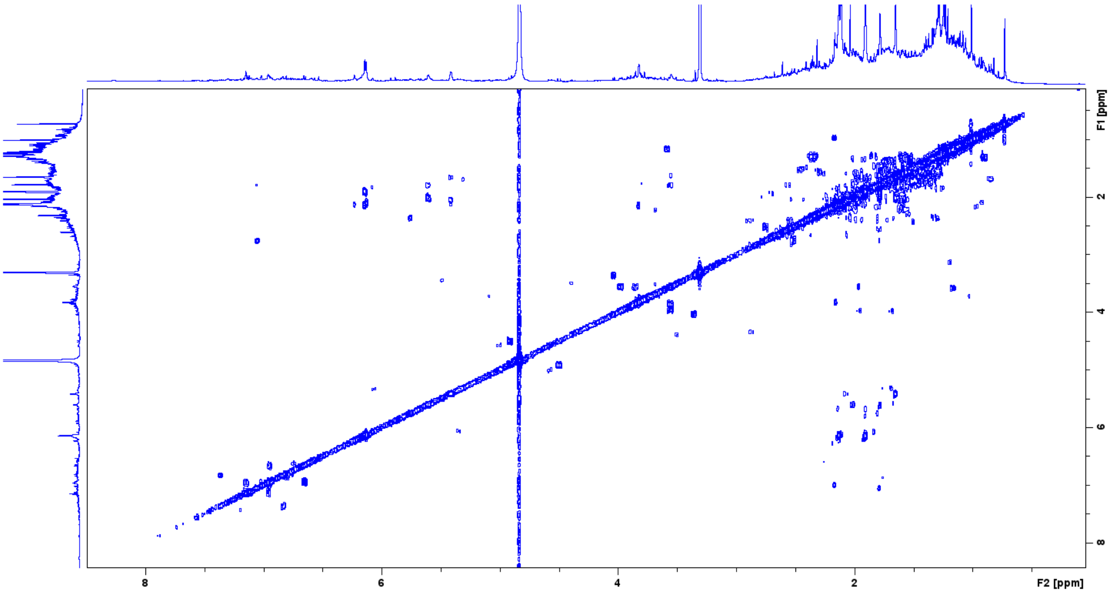

Figure S2. [1] **H-**[1] **H COSY (MeOD) spectrum of the ethanol extract of heartwood**

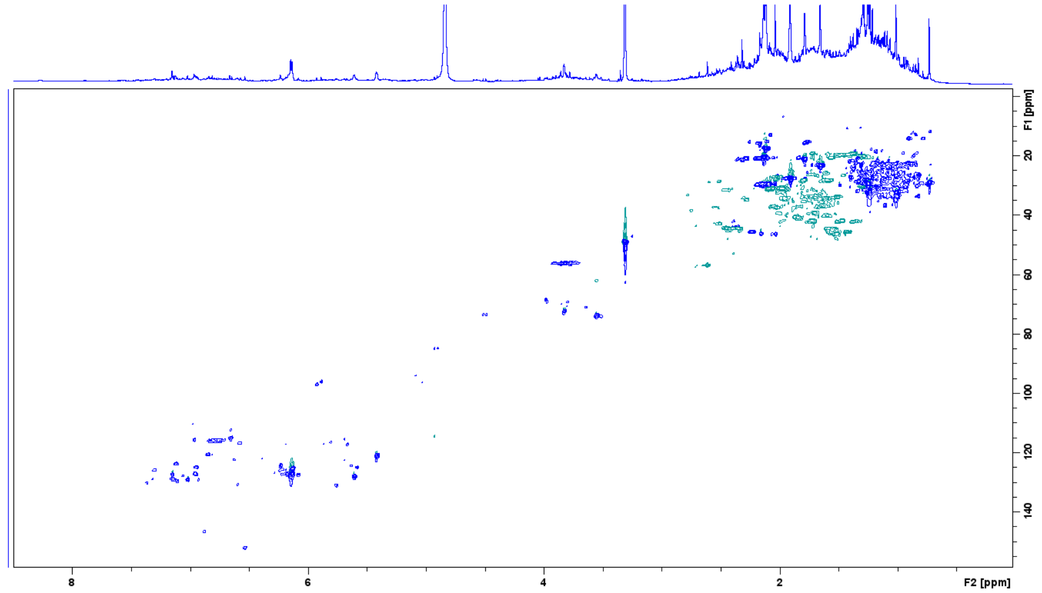

Figure S3. HSQC (MeOD) spectrum of spectrum of the ethanol extract of heartwood

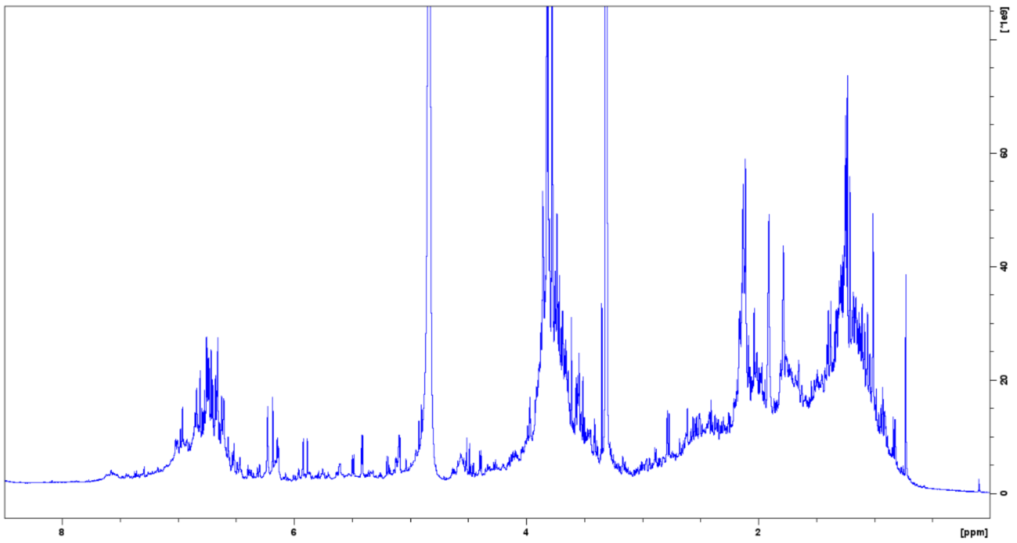

Figure S4. **^{1}H-NMR (400 MHz, MeOD) spectrum of the H_2O extract of heartwood**

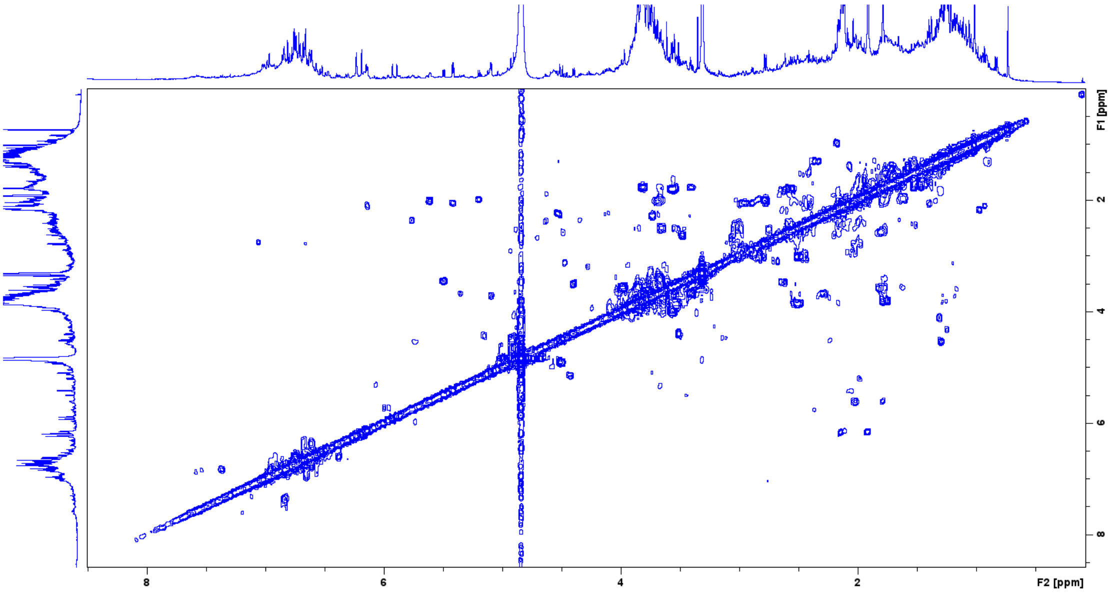

Figure S5. **^{1}H-^{1}H COSY (MeOD) spectrum of the H_2O extract of heartwood**

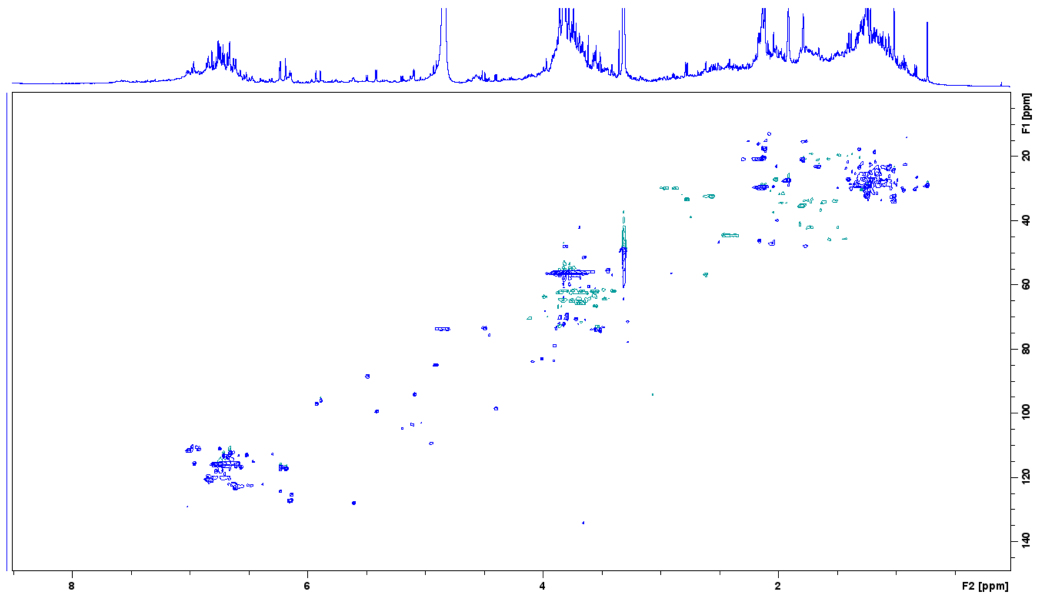

Figure S6. HSQC (MeOD) spectrum of spectrum of the H_2O extract of heartwood

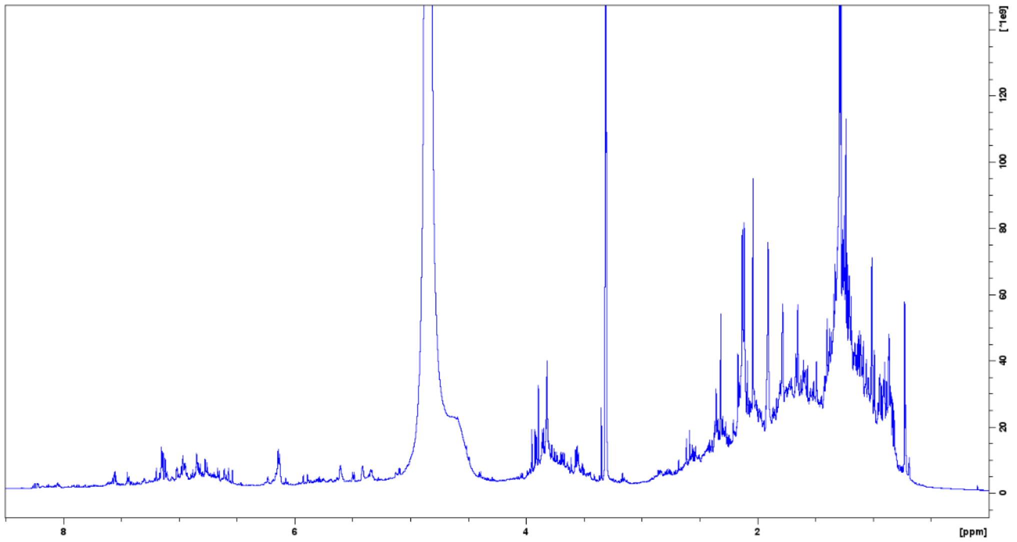

Figure S7. ^{1}H-NMR (400 MHz, MeOD) spectrum of the ethanol extract of sapwood

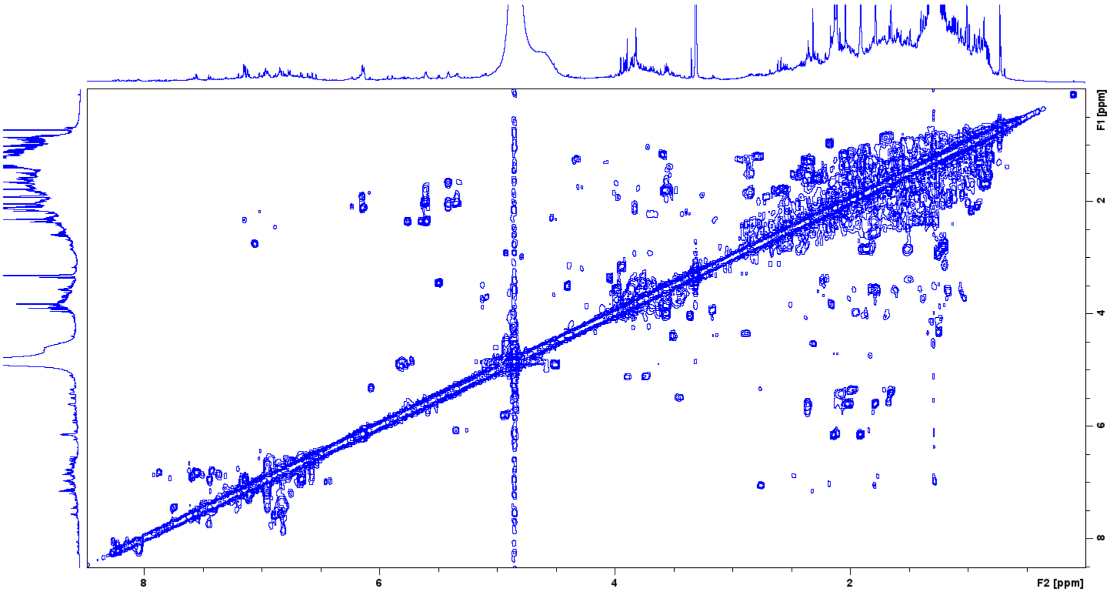

Figure S8. ^{1}H-^{1}H COSY (MeOD) spectrum of the ethanol extract of sapwood

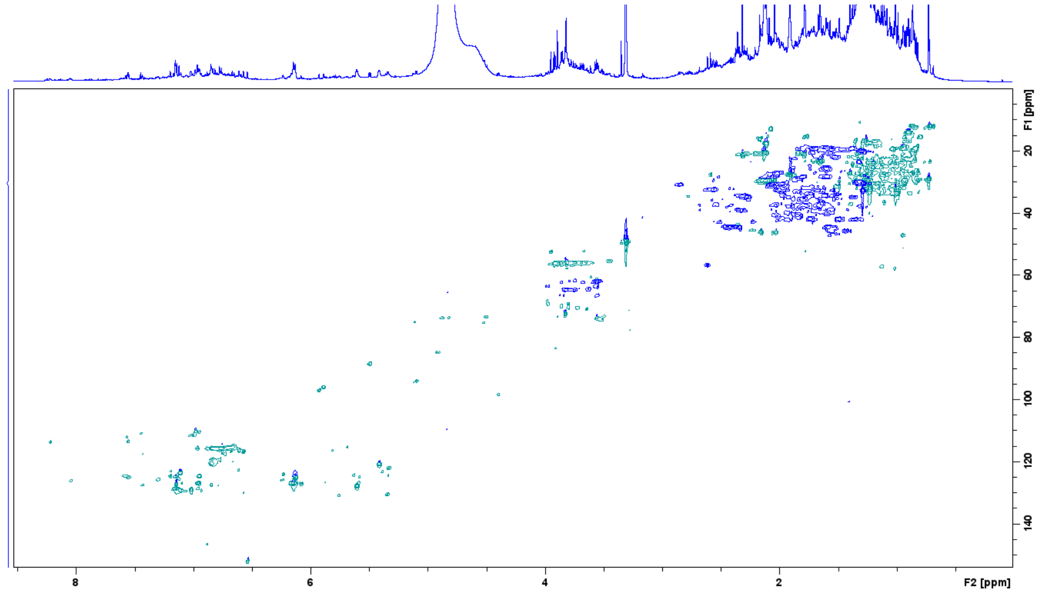

Figure S9. HSQC (MeOD) spectrum of spectrum of the ethanol extract of sapwood

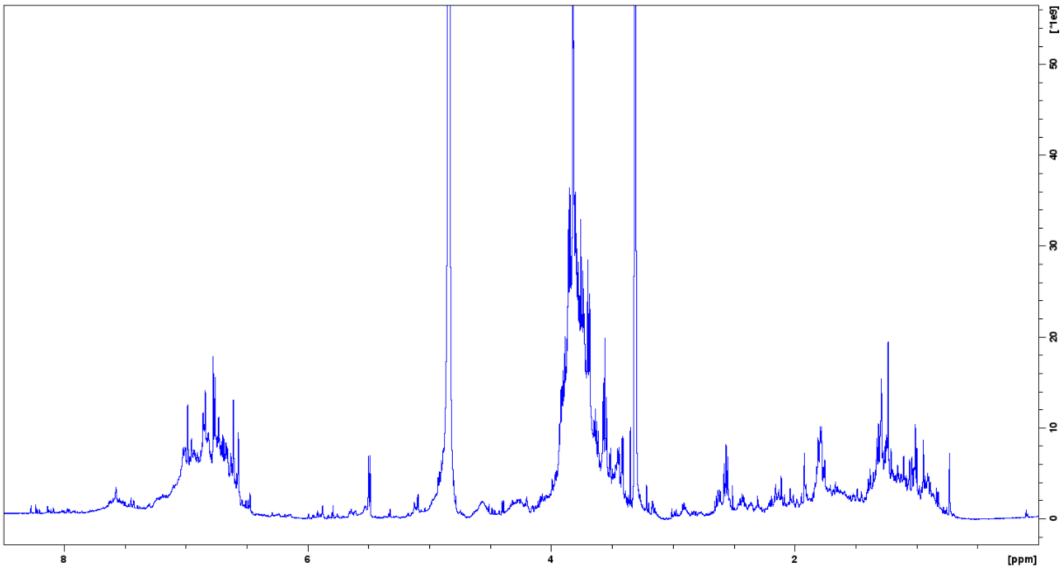

Figure S10. **^{1}H-NMR (400 MHz, MeOD) spectrum of the H_2O extract of sapwood**

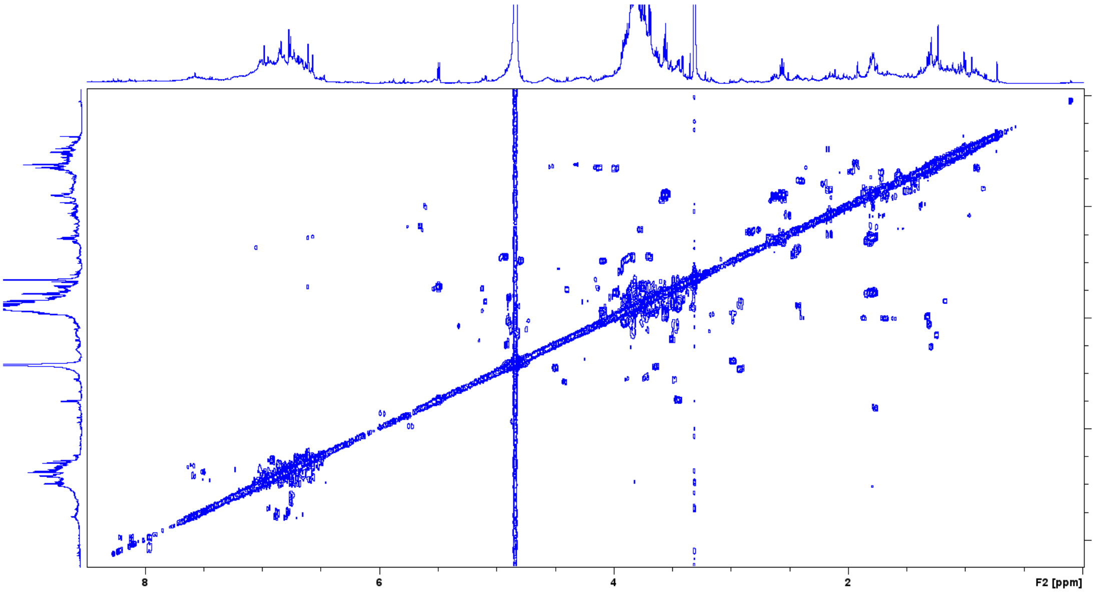

Figure S11. **^{1}H-^{1}H COSY (MeOD) spectrum of the H_2O extract of sapwood**

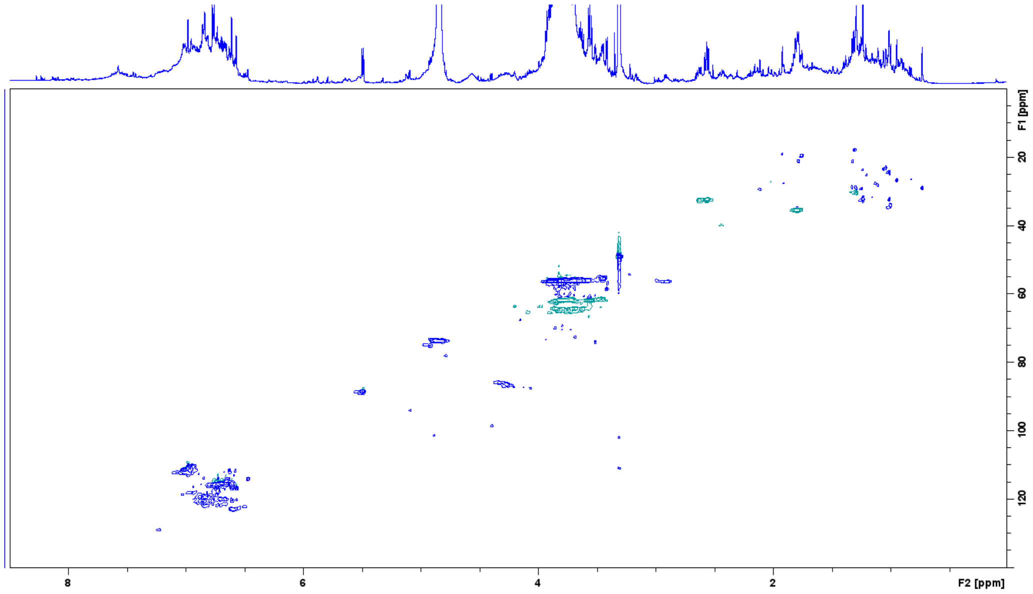

Figure S12. HSQC (MeOD) spectrum of spectrum of the H_2O extract of sapwood

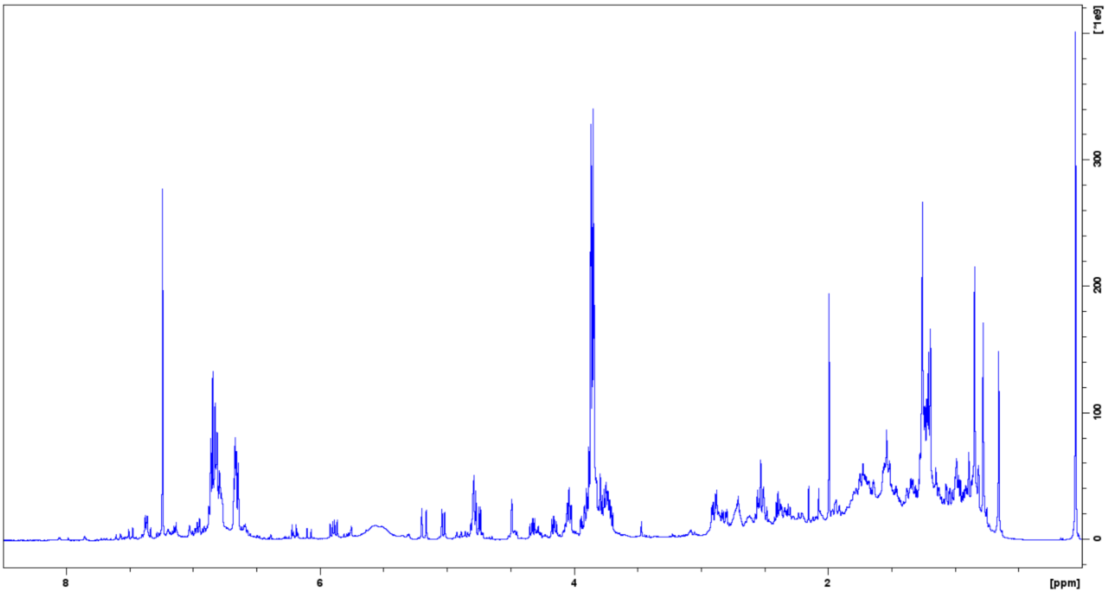

Figure S13. ^{1}H-NMR (400 MHz, $CDCl_3$) spectrum of the resin

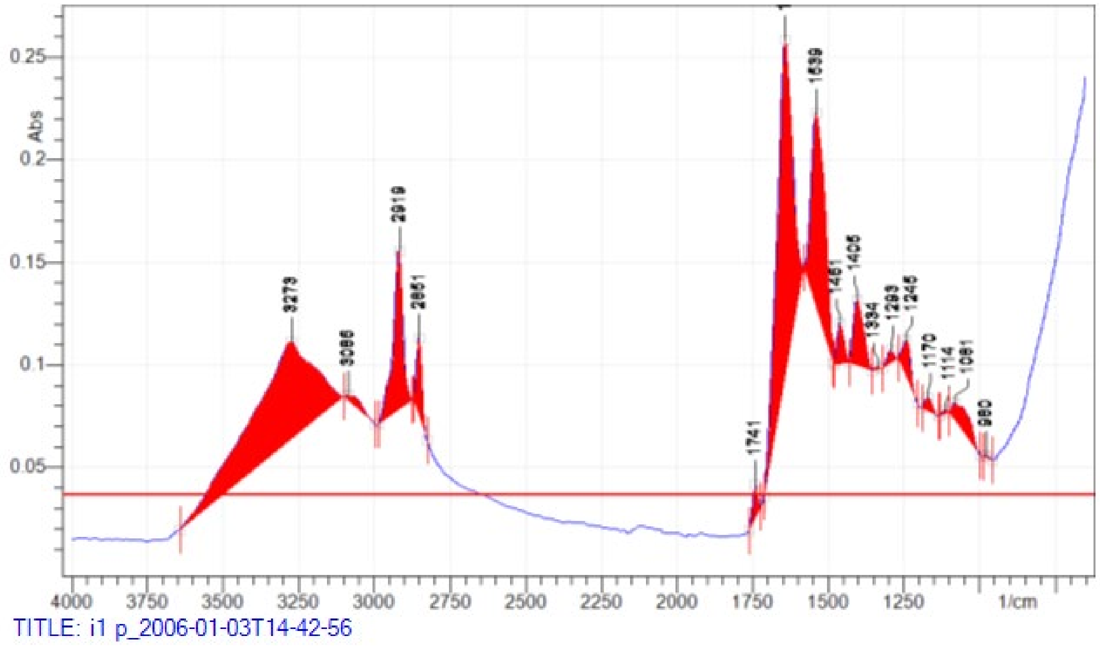

Figure S14. A representative spectrum of normalized and averaged FT-IR spectra on day 14 post-lesion for healthy skin. Changes in band frequency, intensity, and shape were observed across the spectral regions 3700-3000 cm⁻¹, 3000-2850 cm⁻¹, and 1800-800 cm⁻¹.

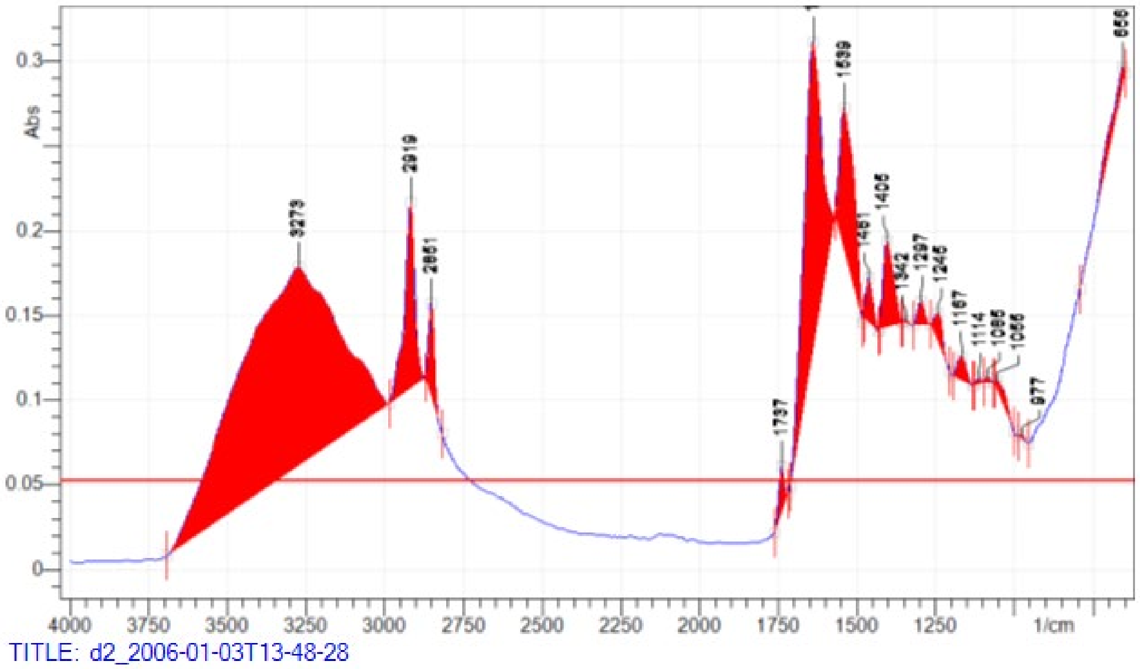

Figure S15. A representative spectrum of normalized and averaged FT-IR spectra on day 14 post-lesion for the control group (untreated mice). Changes in band frequency, intensity, and shape were observed across the spectral regions 3700-3000 cm⁻¹, 3000-2850 cm⁻¹, and 1800-800 cm⁻¹.

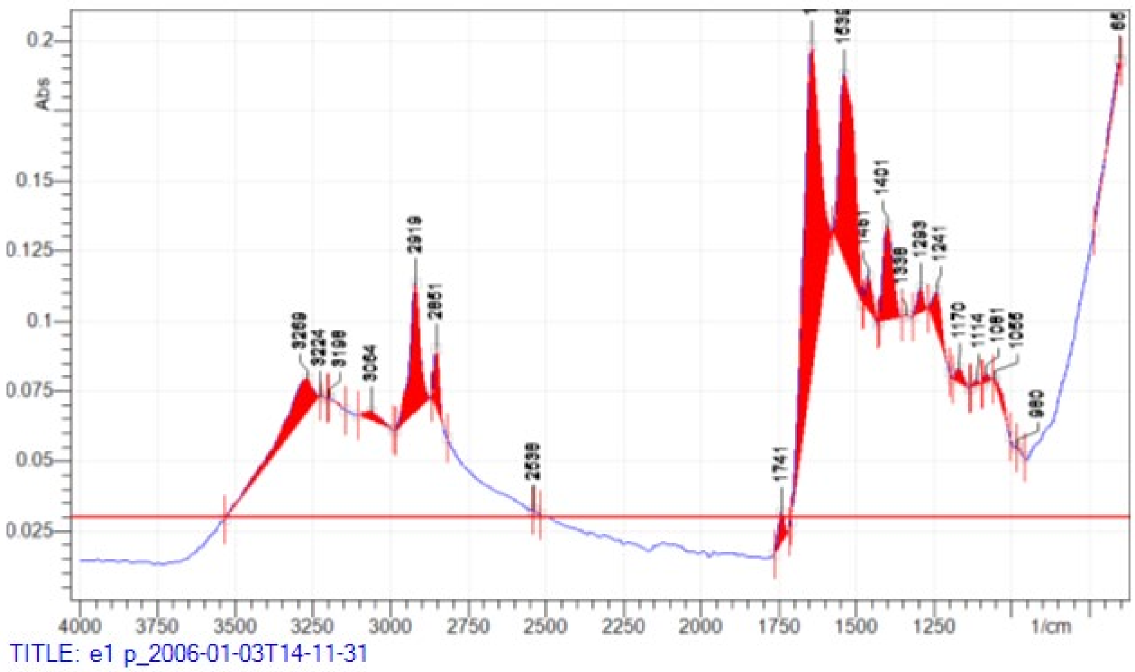

Figure S16. A representative spectrum of normalized and averaged FT-IR spectra on day 14 post-lesion for the vehicle control group (mice treated only with excipients. Changes in band frequency, intensity, and shape were observed across the spectral regions 3700-3000 cm⁻¹, 3000-2850 cm⁻¹, and 1800-800 cm⁻¹.

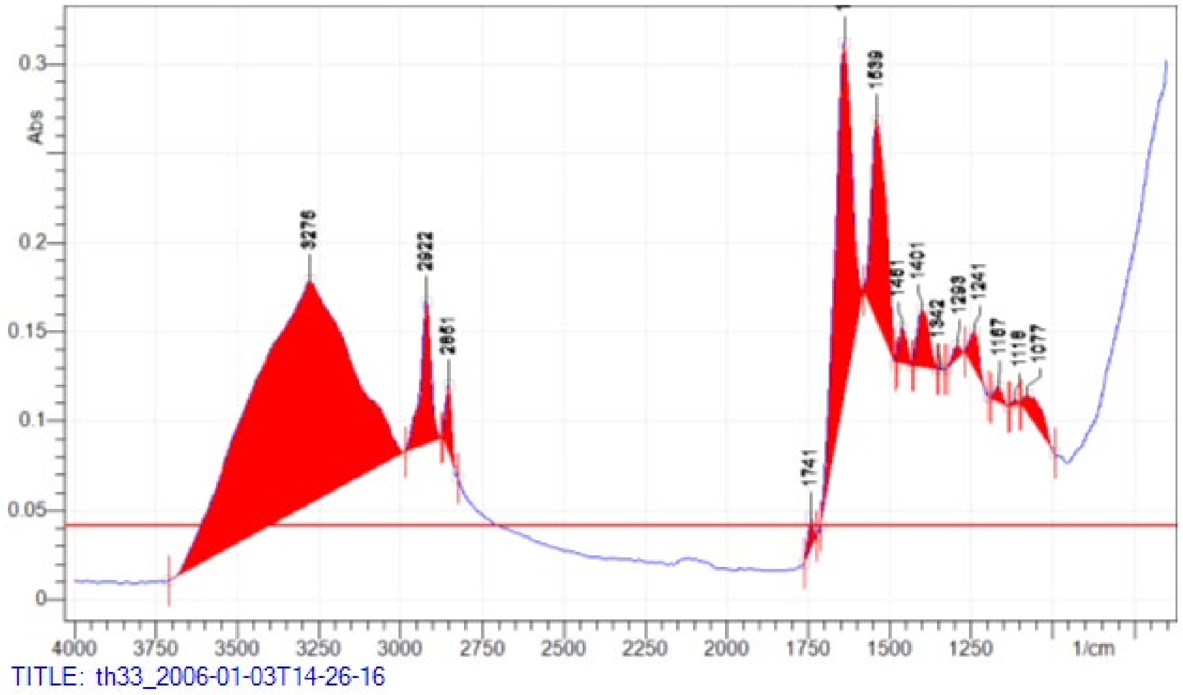

Figure S17. A representative spectrum of normalized and averaged FT-IR spectra on day 14 post-lesion for the 5% w/w resin gel. Changes in band frequency, intensity, and shape were observed across the spectral regions 3700-3000 cm⁻¹, 3000-2850 cm⁻¹, and 1800-800 cm⁻¹.

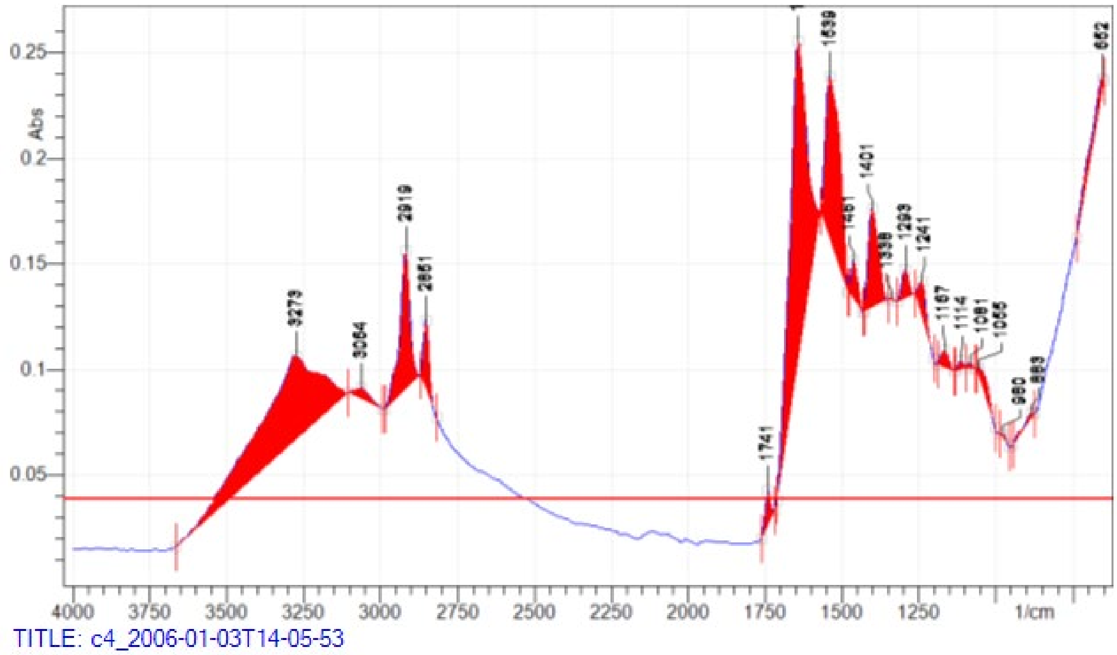

Figure S18. A representative spectrum of normalized and averaged FT-IR spectra on day 14 post-lesion for the 10% w/w resin gel. Changes in band frequency, intensity, and shape were observed across the spectral regions 3700-3000 cm^{-1}, 3000-2850 cm^{-1}, and 1800-800 cm^{-1}.

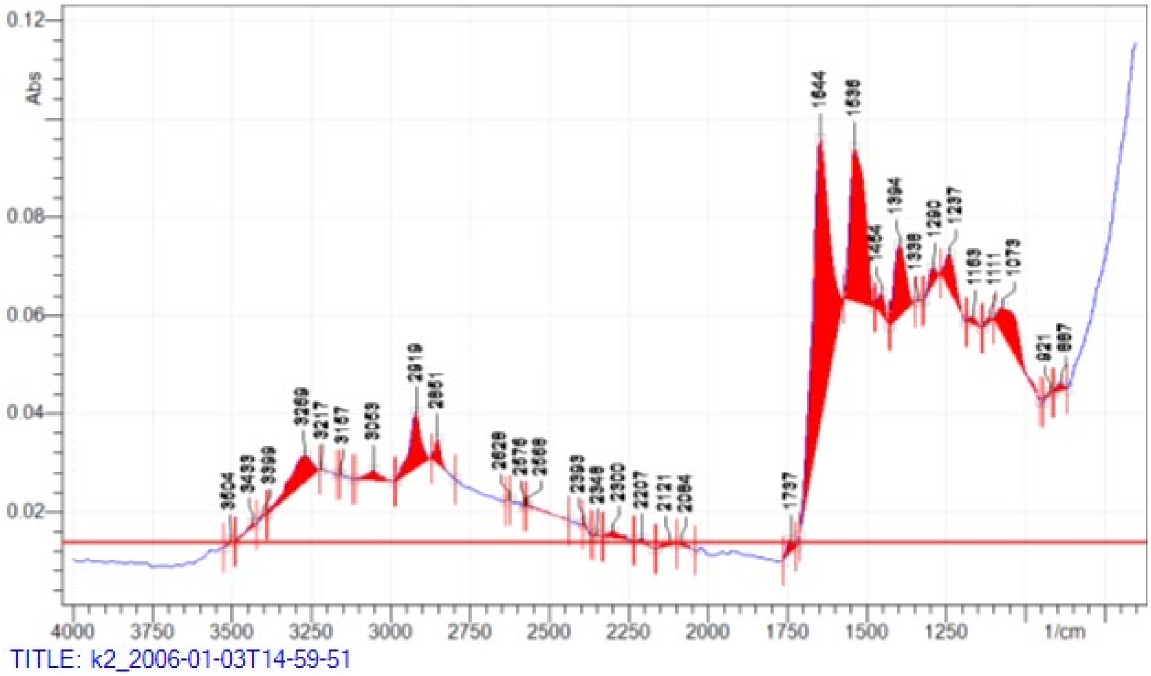

Figure S19. A representative spectrum of normalized and averaged FT-IR spectra on day 14 post-lesion for the 1% w/w sapwood extract gel. Changes in band frequency, intensity, and shape were observed across the spectral regions 3700-3000 cm^{-1}, 3000-2850 cm^{-1}, and 1800-800 cm^{-1}.

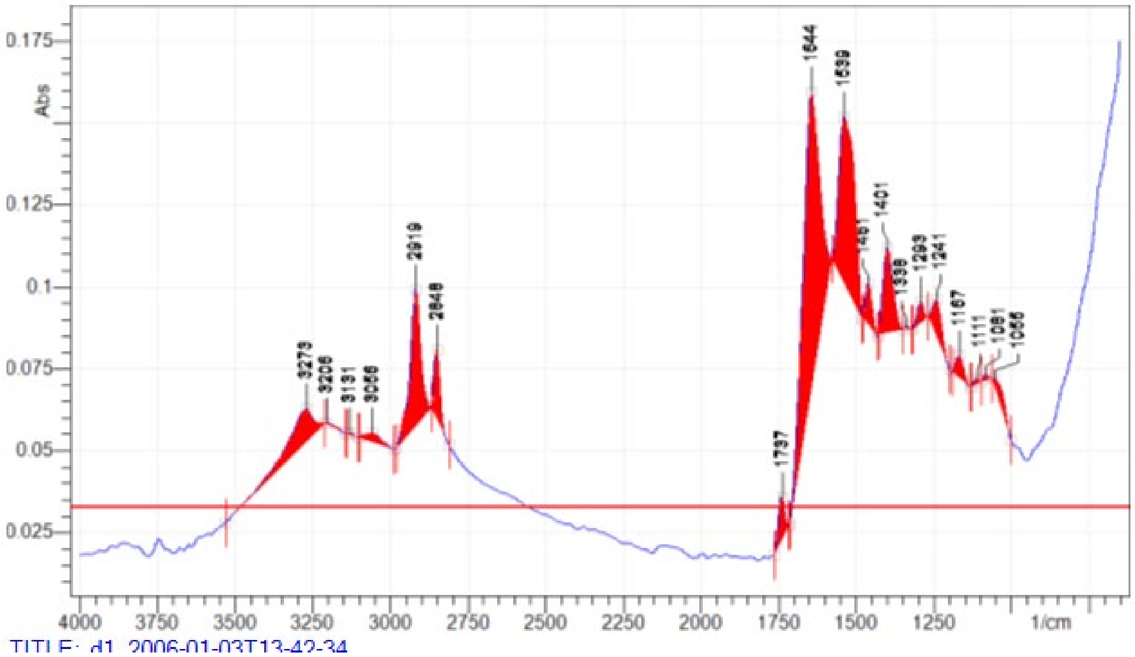

Figure S20. A representative spectrum of normalized and averaged FT-IR spectra on day 14 post-lesion for the 1% w/w sapwood extract gel. Changes in band frequency, intensity, and shape were observed across the spectral regions 3700-3000 cm⁻¹, 3000-2850 cm⁻¹, and 1800-800 cm⁻¹.

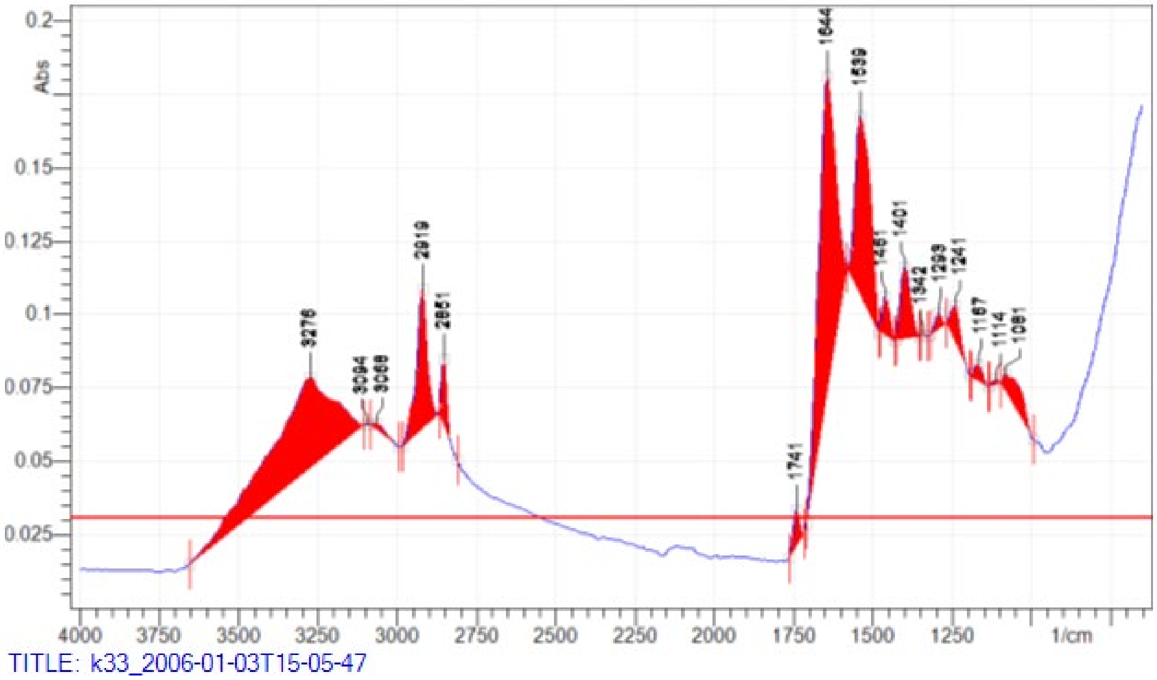

Figure S21. A representative spectrum of normalized and averaged FT-IR spectra on day 14 post-lesion for the 0.5% w/w heartwood extract gel. Changes in band frequency, intensity, and shape were observed across the spectral regions 3700-3000 cm⁻¹, 3000-2850 cm⁻¹, and 1800-800 cm⁻¹.

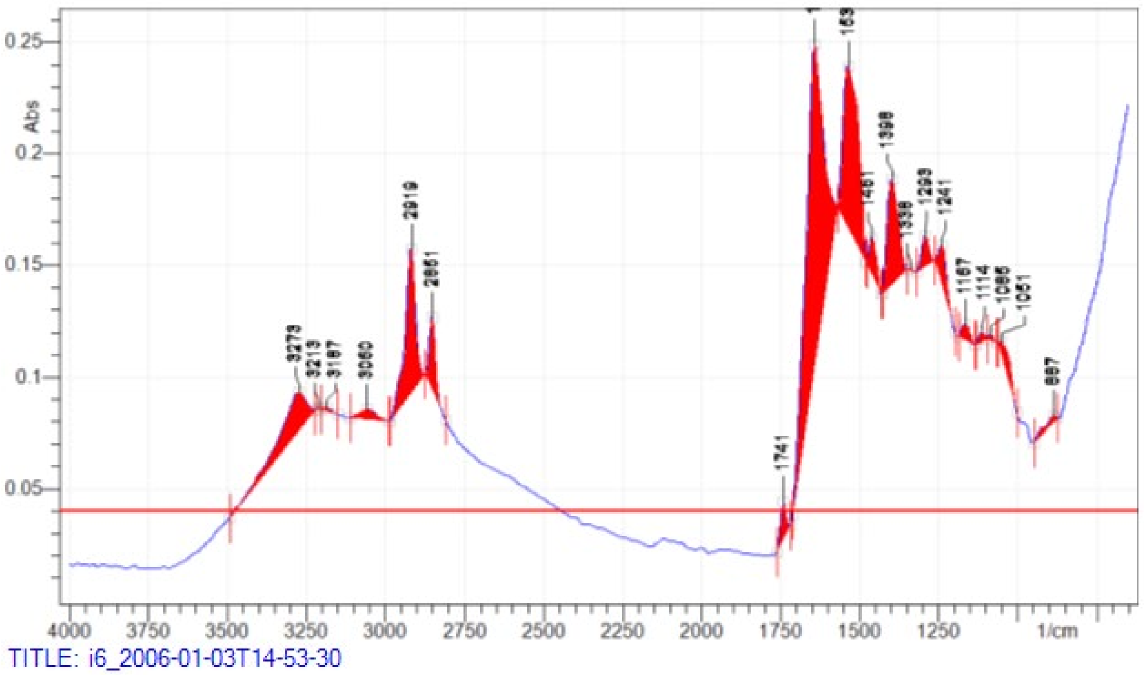

Figure S22. A representative spectrum of normalized and averaged FT-IR spectra on day 14 post-lesion for the 1% w/w heartwood extract gel. Changes in band frequency, intensity, and shape were observed across the spectral regions 3700-3000 cm⁻¹, 3000-2850 cm⁻¹, and 1800-800 cm⁻¹.
